# Extraversion and Neuroticism on College Freshmen's Depressive Symptoms During the COVID-19 Pandemic: The Mediating Role of Social Support

**DOI:** 10.3389/fpsyt.2022.822699

**Published:** 2022-03-07

**Authors:** Tengxu Yu, Jinsheng Hu

**Affiliations:** Liaoning Normal University, Dalian, China

**Keywords:** extraversion, neuroticism, depressive symptoms, social support, multiple mediating effects

## Abstract

**Objective:**

This study aims to explore the direct effects of extraversion and neuroticism on college freshmen's depressive symptoms and their indirect effects via social support under the background of COVID-19.

**Method:**

A total of 3,563 college freshmen were surveyed using the extraversion and neuroticism scales of the Chinese version of the Eysenck Personality Questionnaire (EPQ), the Chinese version of the Beck Depression Inventory-II (BDI-II), and the Social Support Rating Scale (SSRS). Partial correlation analyses and regression analyses were used.

**Results:**

(1) Extraversion had a significant and negative predictive effect on depressive symptoms, while neuroticism positively predicted depressive symptoms. The interaction effect between extraversion and neuroticism on depressive symptoms was also significant. Neuroticism had a greater impact on depressive symptoms in college freshmen with low levels of extraversion. (2) Furthermore, social support partially mediated the relationships between extraversion and depressive symptoms, and neuroticism and depressive symptoms.

**Conclusions:**

Extraversion and neuroticism can directly affect college freshmen' s depressive symptoms, and have indirect effects via the mediating role of social support. Additionally, extraversion interacts with neuroticism, and extraversion plays a protective role in the effect of neuroticism on depressive symptoms. These findings help identify college freshmen at high risk of depression and design effective prevention or intervention measures for them.

## Introduction

Since the World Health Organization (WHO) declared the outbreak of the novel coronavirus disease (COVID-19) on January 30, 2020, this pandemic continues to spread at breakneck speed and has induced severe psychological trauma to people worldwide including China. To limit the spread among students in high schools and colleges, the Ministry of Education in China limited offline teaching and to some extent promoted online teaching. The restrictions on social face-to-face contact, such as meeting friends or participating in extracurricular activities, may indirectly lead to more negative emotions and stress to Chinese students. Several studies have found the negative effect of COVID-19-related stressors on mental health, such as an increase in depressive and anxiety symptoms (1–3).

As one of the major mental disorders which greatly endanger human health, depression is characterized by high rates of morbidity, recurrence, and also has a high probability of causing disability and death (4, 5). As for college freshmen, the transition from high school to college could easily cause maladjustment that lead to an increasing risk of mental health problems including depression (6, 7). Therefore, COVID-19 epidemic pressure and the maladjustment caused by transition may interact and double the risk of depression among college freshmen. Despite this, little attention has been given to the mental health of college freshmen under the background of COVID-19 (8). As the fight against COVID-19 epidemic will not end in the short term, it is necessary to explore the antecedents (both protective and risk factors) of the onset of depressive symptoms among college freshmen and further design specific and effective intervention for them within COVID-19. Therefore, to contribute to this gap, we selected two personality traits: extraversion and neuroticism and then tested how they may, respectively, mitigate and exacerbate depressive symptoms in college freshmen, with the mediating effect of social support.

### Extraversion and Depressive Symptoms

Extraversion is usually associated with positive characteristics, such as sensation-seeking, venturesome, active, surgent, sociable, lively, and assertive. Therefore, this personality trait is found to have an inverse correlation with mental health (9, 10). People with higher extraversion levels tend to be more positive than the lower extraversion level people. The latter may have little interest in the outside world (11). The influence of the COVID-19 epidemic pressure on individuals' mental health will likely vary depending on their extraversion level. People with higher extraversion scores participant in more social activities and seek more help and comfort from the outside world than those with lower extraversion scores. Thus, extraversion gives some protection to individuals against the epidemic pressure, and then reduce their depressive symptoms. Several studies have revealed the inverse correlation between extraversion and depressive symptoms (12–15). For example, Jylhä and Isometsä (10) measured extraversion and depressive symptoms via the Eysenck Personality Inventory (EPI) and Beck Depression Inventory (BDI), respectively and found a significant negative correlation between them. Recently, another study also found that higher levels of extraversion were protective against depressive mood (12).

### Neuroticism and Depressive Symptoms

On the contrary, neuroticism is characterized by being anxious, depressed, guilty, tense, irrational, shy, moody, emotional, and having low self-esteem. This personality trait is often found to be a risky factor for mental health (12, 16). Life stress exposure is thought to be related with neuroticism. Individuals with higher levels of neuroticism could have more negative feelings, such as depressive and nervous, when under stressors (17). We deduce that neuroticism increases depressive symptoms during the COVID-19 pandemic. Empirically, the positive association between neuroticism and depressive symptoms has been confirmed by several studies (18–20). An early longitudinal study found that levels of neuroticism positively predicted the risks for major depression (16). Vinograd et al. (18) again found that neuroticism was associated with the severity of depressive symptoms in a recent horizontal study.

Extraversion and neuroticism are negatively and positively correlated with depressive symptoms, respectively. Furthermore, a few studies explored the interaction between extraversion and neuroticism on depressive symptoms. For example, Grav et al. (13) examined the relationship between extraversion, neuroticism, and depressive symptoms in western culture (Norway), and the results revealed a significant interaction between extraversion and neuroticism on depressive symptoms. To be specific, low extraversion increased the predictive effect of neuroticism on depressive symptoms. Boudouda and Gana (12) also found a significant “extraversion × Neuroticism” interaction in relation to depressive mood in a non-Western culture. They further explained that extraversion played a protective role in the relation between neuroticism and depressive symptoms.

### Social Support as a Mediator

Social support can be defined as a series of support sources that individuals can access through social relationships. These support sources include family, friends, colleagues and community (21). Previous studies found that social support was related to both personality traits and depressive symptoms. Poor social support has been positively associated with depressive symptoms (22, 23). Moreover, researchers found that high levels of extraversion correlated with more social support (24), while individuals with high levels of neuroticism are more likely to have less social support (24, 25). Therefore, social support may play an important role in the relationship between personality traits and depressive symptoms. Going further, interpersonal theories of depression posit that depressed individuals tend to interact with others in a way that elicits rejection, which means the depressed individuals have less social support and increases their risk for future depression (26). In line with this theoretical perspective, social support are more likely to play a mediating other than moderating role in the relationship between personality and depressive symptoms. A few empirical studies have also proved that social support had mediation effects between personal traits and depressive symptoms (25, 27, 28). For example, Fang et al. (28) found that perceived social support mediated the narcissism–depression pathway. Based on the previous studies above, we assumed that social support would mediate the relationship between extraversion and depressive symptoms, and the relationship between neuroticism and depressive symptoms.

### The Present Study

**Figure 1:**
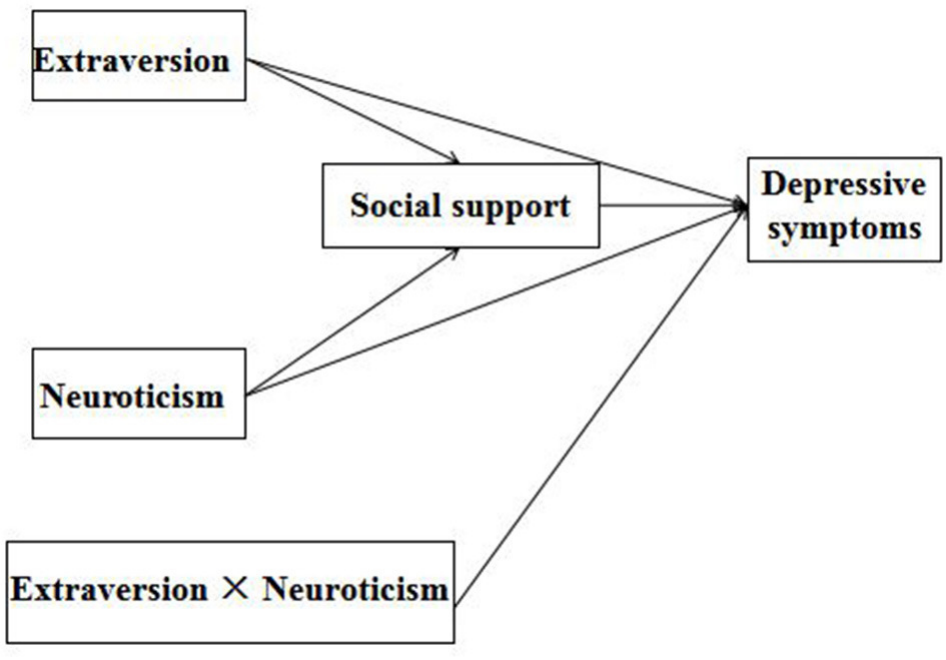
Proposed conceptual model.

First, we wanted to verify the predictive effects of extraversion, neuroticism, and the interaction between them on depressive symptoms. Second, the study aimed to test whether social support would mediate the relationship between extraversion and depressive symptoms, and the relationship between neuroticism and depressive symptoms (see Figure 1). Based on the literature review, two hypotheses were proposed as follows:

*Hypothesis 1*. Extraversion and neuroticism would negatively and positively predict depressive symptoms. The interaction between the two personality traits would also predict depressive symptoms. Specifically, the predictive effect of neuroticism would be stronger for college freshmen with lower levels of extraversion, than those with higher scores in extraversion.

*Hypothesis 2*. Social support would mediate the relationship between extraversion and depressive symptoms, and the relationship between neuroticism and depressive symptoms.

## Method

### Participants

The datasets for this study were collected during September and October 2021, when the college freshmen had just entered university. All the first-year students (3,680) of one university in China's Liaoning province were recruited, and completed four questionnaires on a specially designed mobile app. We eliminated 117 invalid questionnaires based on the inclusion and exclusion criteria (participants completed one item at least for 2 s, so the participants who completed each questionnaire too fast, thus, completed every item for <2 s were excluded). Thus, we finally retained 3,563 valid questionnaires with 1,120 males and 2,443 females.

### Measures

#### Extraversion and Neuroticism

Extraversion and neuroticism were evaluated by the Eysenck Personality Questionnaire (EPQ), which was developed by Eysenck (29). We used the Chinese version of the EPQ, revised by Gong (30). The extraversion (E) subscale consists of 21 items (e.g., “Do you have many different hobbies?”), and the neuroticism (N) subscale consists of 24 items (e.g., “Do you get excited easily?”). Participants answered “yes” or “no,” which each take the value of 1 or 0, respectively. A higher score indicated a higher level of each trait. In this study, Cronbach' s α was 0.83 and 0.89 for the E and N scales, respectively. The total scores of the two subscales were used as indicators of extraversion and neuroticism, respectively.

#### Depressive Symptoms

Depressive symptoms were evaluated by the Beck Depression Inventory-II [BDI-II (31)]. BDI-IIconsists of 21 items related to symptoms of depression. Each question consists of four statements scored from 0 to 3, with higher scores implying increasing symptoms severity. People who score 0–13 are considered free of depression; people scoring 14–19 are seen as “mildly”; 20–28 as “moderately”; and 29–63 as “severely” depressive (32–34). In this study, Cronbach' s α was 0.89.

#### Social Support

Social support was evaluated by the Social Support Rating Scale (SSRS), compiled by Xiao (35). SSRS contains 10 questions divided into three dimensions: subjective support (questions 1, 3–5, e.g., “How many close friends who can provide support and help, do you have?”), objective support (questions 2, 6–7, e.g., “How many sources, from which you got comfort and care when in emergency, do you have?”) and utilization of support (questions 8–10, e.g., “To what extent are you willing to ask for help from others?”). Answers 1–4 to questions 1–4 and 8–10 correspond to 1–4 points, respectively. For question 5, according to the support degree of the a–d options, participants answered 1–4 to each options to obtain 1–4 points. As for questions 6 and 7, the numbers of support sources ranging from 1 to 9 is exactly equal to the scores one could obtain. A higher score indicates a higher level of social support. In this study, Cronbach' s α for SSRS was 0.83.

### Procedure

The college freshmen entered one classroom in batches. First, research assistants explained the procedure and issues that needed attention, and then told the students how to enter the mobile app via WeChat. The participants completed the four online questionnaires on the spot. Ethical approval was obtained from the Ethics Committee for Scientific Research at the corresponding author's university.

### Data Analysis

We used SPSS 22.0 and Mplus 7.0 to organize and analyze the data. The correlations between each variables were analyzed via SPSS 22.0. Then structural equation modeling (SEM) through Mplus 7.0 was used to analyze the proposed model, with all regression coefficients tested by the bias-corrected percentile Bootstrap method. We tested the mediation and moderating effects in the proposed model by estimating their 95% confidence interval (CI) with 1,000 resampled samples. Age and gender were controlled in the correlation and regression analyses.

## Results

### Common-Method Biases Test

Firstly, we undertook the Harman single factor test to examine the common method biases. The results showed that 14 factors had eigenvalues >1, and the first factor explained 18.39% of the total variance, which did not exceed the critical value of 40% (36).

### Preliminary Analyses and Descriptive Data

Of the 3,563 freshmen, 2,887 (81.03%) were free of depression; and 352 (9.88%), 243 (6.82%), 81 (2.27%) were mildly, moderately, and severely depressive, respectively. Table 1 showed the means, standard deviations, and correlation matrix of each variable. As shown in Table 1, depressive symptoms were positively associated with neuroticism (*r* = 0.69, *p* < 0.001); negatively associated with extraversion (*r* = −0.33, *p* < 0.001) and social support (*r* = −0.35, *p* < 0.001).

**Table 1:** Descriptive statistics and correlations among variables.

	**M**	**SD**	**1**	**2**	**3**	**4**
Extraversion	11.58	4.71	1			
Neuroticism	9.42	4.71	−0.25^***^	1		
Depressive symptoms	7.68	7.86	−0.33^***^	0.69^***^	1	
Social support	42.72	8.50	0.40^***^	−0.32^***^	−0.35^***^	1

*** *p <0.001*.

### Mediation Effect Analysis

**Figure 2:**
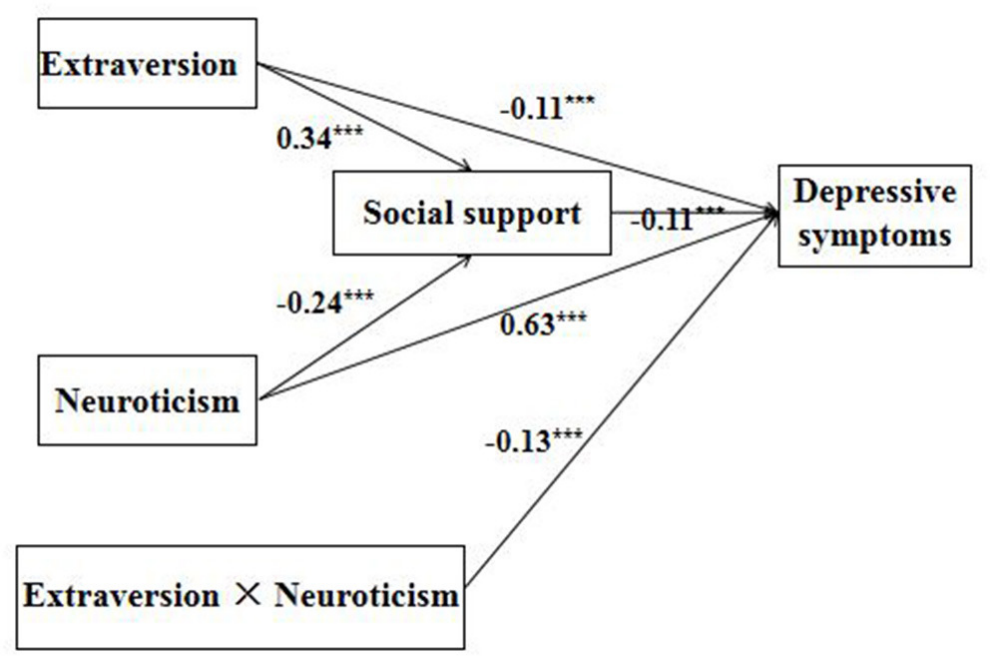
Path rnodel of the proposed conceptual model. ^***^p < 0.001.

All the 3,563 participants were analyzed in the multiple mediation model. Figure 2 shows the proposed model's great fit (RMSEA = 0.035, SRMR = 0.010, CFI = 0.996, TLI = 0.986) based on field threshold standards. In the model, the negative predictive effect of extraversion on depressive symptoms was significant (β = −0.11, *p* < 0.001); the positive predictive effect of neuroticism on depressive symptoms was also significant (β = 0.63, *p* < 0.001), while social support had a significant negative predictive effect on depressive symptoms (β = −0.11, *p* < 0.001). Extraversion and neuroticism negatively interacted in their relation to depressive symptoms (β = −0.13, *p* < 0.001). Moreover, extraversion positively predicted social support (β = 0.34, *p* < 0.001); and neuroticism negatively predicted social support (β = −0.24, *p* < 0.001). In addition, the upper and lower bounds of the bootstrapped 95% CI for the mediating effects of social support did not include 0, indicating the mediating effects in the relation between extraversion and depressive symptoms (indirect effect = −0.035, SE = 0.005, 95% CI = [−0.044, −0.027]) and the relation between neuroticism and depressive symptoms (indirect effect = 0.025, SE = 0.004, 95% CI = [0.019, 0.032]) were both significant. The total effects of extraversion and neuroticism on depressive symptoms were −0.145 and 0.655, respectively. The effect size was based on the ratio of the mediating effect to the total effect. Therefore, the effect size of social support in the relationship between extraversion and depressive symptoms was 24.14%, while the effect size in the relationship between neuroticism and depressive symptoms was 3.82%.

**Figure 3:**
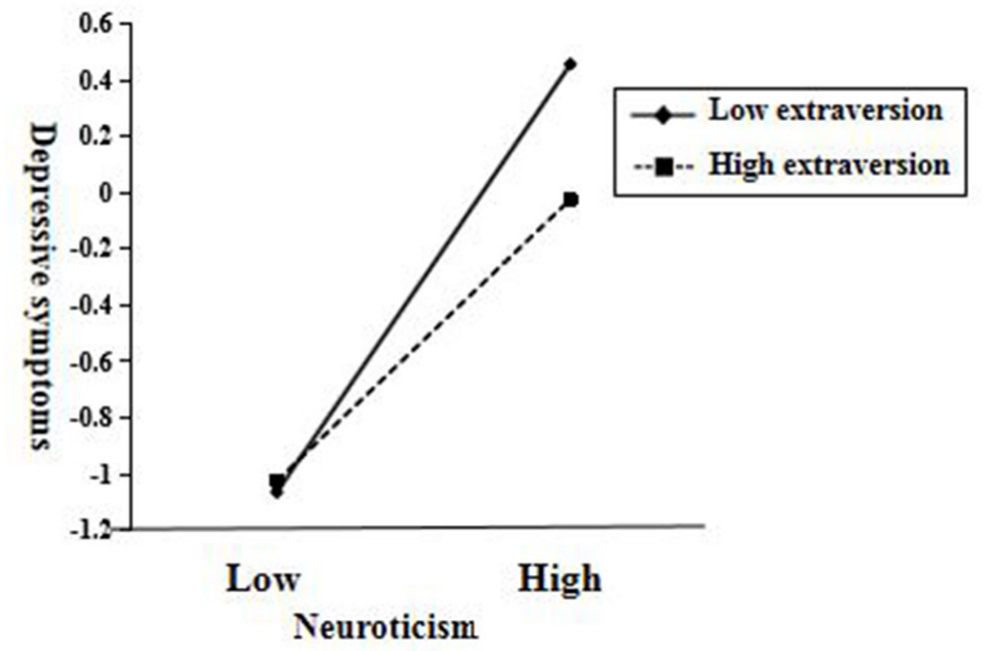
Interaction plot between neuroticism and extraversion on depressive symptoms.

The interaction effect was shown in Figure 3. Extraversion played a moderating role in the effect of neuroticism on depressive symptoms. For freshmen with low extraversion (M_extraversion_ - SD_extraversion_), the positive predictive effect of neuroticism was bigger (β = 0.75, *p* < 0.001); while for freshmen with high extraversion (M_extraversion_ + SD_extraversion_), the positive predictive effect of neuroticism was smaller (β = 0.50, *p* < 0.001).

### Considering Alternative Models

Although results have generally provided strong support for our current model, the possible alternative models should also be considered and tested. The reason for considering alternative models is that we want to identify whether there should be the interaction item and whether the interaction item points to social support or depressive symptoms (the current model (named Model 3) with the interaction item pointing to depressive symptoms). Therefore, we chose two possible alternative models. One is the multiple mediation model without the interaction item (named Model 1) and another is the multiple mediation model with the interaction item pointing to social support (named Model 2). We further compared the fitting parameters of these three models and results were shown in Table 2. The fitting parameters of Model 2 was not good and thus excluded. The fitting parameters of Model 1 was as good as those of the current model 3. However, as the interaction effect between extraversion and neuroticism on depressive symptoms has been confirmed in previous studies (12, 13), we chose Model 3 rather than Model 1 for the present study.

**Table 2:** Comparison of alternative models.

**Model**	**RMSEA**	**CFI**	**TLI**	**SRMR**
1	0.033	0.998	0.990	0.009
2	0.102	0.968	0.883	0.021
Current model 3	0.035	0.996	0.986	0.010

## Discussion

In line with one previous study, whose results showed that mild, moderate, and severe depressive symptoms of college students accounted for 8.9, 5.4, and 2.4%, respectively (37), this study revealed similar prevalence of depression for college freshmen: the prevalence for moderate and severe depression, with mild, moderate, and severe depressive symptoms of college freshmen accounted for 9.88, 6.82, and 2.27%, respectively. Based on previous empirical studies, we then constructed a multiple mediating model in which the two personality traits (extraversion and neuroticism) and depressive symptoms were entered as independent and dependent variables, respectively, with social support as a mediator. The study set out to investigate another two questions. First, do extraversion and neuroticism have respective and interactive effects on college freshmen' s depressive symptoms? Second, how do the two personality traits affect depressive symptoms via social support? The results could deepen our understanding of the relationship between personality traits and depressive symptoms at the theoretical level. The results also have practical implication for the intervention of college freshmen's depressive symptoms under COVID-19.

### Extraversion, Neuroticism, and Depressive Symptoms

This study found that both extraversion and neuroticism could directly affect college freshmen's depressive symptoms. The results are in line with previous studies (10–13). To be specific, extraversion is always associated with positive emotions, energy and relationship, all of which can protect individuals from depressive symptoms (38, 39), while neuroticism can lead to worry, fear, nervous and other negative emotions, which can further increase individuals' depressive symptoms (40–42).

Moreover, previous studies demonstrated that extraversion and neuroticism not only had respective effects, but also interacted on individuals' mental health, especially during the COVID-19 pandemic (12, 43). As for depression, low extraversion and high neuroticism are both risk factors, and their respective effects can be enhanced by each other (13). In line with these results above, this study also found an interaction effect between extraversion and neuroticism on college freshmen's depressive symptoms. For one thing, high extraversion played a protective role and could weaken the effect of neuroticism on depressive symptoms; for another, college students with both low extraversion and high neuroticism have a higher risk of depression, and thus should receive more attention for prevention or intervention of depression.

### The Mediating Role of Social Support

Social support is important in the adaptation of college freshmen (44, 45), and closely correlates with both personality traits (46, 47) and depressive symptoms (48, 49). However, existing studies have tended to focus on the direct effects of personality traits on depressive symptoms or the protective role of social support against depressive symptom, whereas few researches focused on the mechanism of social support in the relationship between personality traits and depressive symptoms. This study found that extraversion and neuroticism can not only directly affect depressive symptoms among college freshmen, but also have indirect effects on depressive symptoms through the mediating role of social support. Students with high levels of extraversion are likely to have good social relationships and get more support from others around them (14), while students with high levels of neuroticism are probably not good at social activities and thus lack social support (46). Empirical studies also found that a lack of social support is a vulnerability factor for depressive symptoms (48, 49), hence, extraversion and neuroticism can decrease and increase depressive symptoms by increasing and decreasing social support, respectively.

### Limitations and Practical Implications

There are some limitations to this study. First, the causality between personality traits (extraversion and neuroticism) and depressive symptoms cannot be deduced from the results of this cross-sectional study. Future researchers could adopt a longitudinal design to examine the causal relationship between the two variables. Second, this study aimed to integrate individual variables (personality traits) and environmental variables (social support) influencing students' depressive symptoms. However, only two personality traits, that is, extraversion and neuroticism were included, and social support cannot be on behalf of all environmental variables. Thus, in future researches, other personality traits such as openness and environmental variables like family environment should be included. Third, according to previous studies, Eysenck Personality Questionnaire-Revised Short Form (EPQR-S) was found a better measurement with higher credibility and validity than EPQ under non-Chinese culture (50). In future, Chinese researchers should verify the results of the present study using EPQR-S. Finally, this study collected data only through self-report measures, which may exist in socially desirable bias and other biases, especially during the transition from high school to university. Some college freshmen might not answer truthfully on some sensitive questions, and other college freshmen could experience some maladjustment just before the period of data collection, which can both cause bias. In future studies, therefore, multiple measures, such as semi-structured interviews can be introduced.

Despite these limitations, this study also has some implications. First, in line with previous studies, this study also found the negative and positive predictive effects of extraversion and neuroticism on depressive symptoms. This can help identify students who are at high risk of depression, that is, those with low levels of extraversion and high levels of neuroticism. Second, the results of this study provided one mechanism explaining how personality traits affect depressive symptoms. Specifically, extraversion and neuroticism indirectly affected depressive symptoms through the mediating role of social support. Hence, future prevention or intervention in college freshmen's depressive symptoms can target improving their social support.

## Data Availability Statement

The original contributions presented in the study are included in the article/supplementary material, further inquiries can be directed to the corresponding author/s.

## Ethics Statement

The studies involving human participants were reviewed and approved by Ethics Committee of Liaoning Normal University. Written informed consent to participate in this study was provided by the participants' legal guardian/next of kin.

## Author Contributions

JH contributed to the conception of the study and was responsible for the data collection. TY analyzed the data and wrote the draft of the paper. Both authors contributed to the article and approved the submitted version.

## Funding

This research was funded by the grants of National Social Science Fund of China (No. BIA200182).

## Conflict of Interest

The authors declare that the research was conducted in the absence of any commercial or financial relationships that could be construed as a potential conflict of interest.

## Publisher's Note

All claims expressed in this article are solely those of the authors and do not necessarily represent those of their affiliated organizations, or those of the publisher, the editors and the reviewers. Any product that may be evaluated in this article, or claim that may be made by its manufacturer, is not guaranteed or endorsed by the publisher.
